# High-throughput evaluation of T7 promoter variants using biased randomization and DNA barcoding

**DOI:** 10.1371/journal.pone.0196905

**Published:** 2018-05-07

**Authors:** Ryo Komura, Wataru Aoki, Keisuke Motone, Atsushi Satomura, Mitsuyoshi Ueda

**Affiliations:** 1 Division of Applied Life Sciences, Graduate School of Agriculture, Kyoto University, Kitashirakawa Oiwake-cho, Sakyo-ku, Kyoto, Japan; 2 Japan Society for the Promotion of Science, Kitashirakawa Oiwake-cho, Sakyo-ku, Kyoto, Japan; Imperial College London, UNITED KINGDOM

## Abstract

Cis-regulatory elements (CREs) are one of the important factors in controlling gene expression and elucidation of their roles has been attracting great interest. We have developed an improved method for analyzing a large variety of mutant CRE sequences in a simple and high-throughput manner. In our approach, mutant CREs with unique barcode sequences were obtained by biased randomization in a single PCR amplification. The original T7 promoter sequence was randomized by biased randomization, and the target number of base substitutions was set to be within the range of 0 to 5. The DNA library and subsequent transcribed RNA library were sequenced by next generation sequencers (NGS) to quantify transcriptional activity of each mutant. We succeeded in producing a randomized T7 promoter library with high coverage rate at each target number of base substitutions. In a single NGS analysis, we quantified the transcriptional activity of 7847 T7 promoter variants. We confirmed that the bases from −9 to −7 play an important role in the transcriptional activity of the T7 promoter. This information coincides with the previous researches and demonstrated the validity of our methodology. Furthermore, using an *in vitro* transcription/translation system, we found that transcriptional activities of these T7 variants were well correlated with the resultant protein abundance. We demonstrate that our method enables simple and high-throughput analysis of the effects of various CRE mutations on transcriptional regulation.

## Introduction

Transcriptional regulation by cis-regulatory elements (CREs) such as promoter [1], enhancer [2], and other elements [3] are one of the mechanisms for controlling gene expression. CREs are typically non-coding DNA regions and regulate transcription of downstream genes by binding regulatory factors [4]. Mutations in CREs are one of the causes of phenotypic changes distinguishing species [5,6]. In addition, mutations in CREs are frequent causes of human diseases [7,8]. Thus, studies involving mechanisms of controlling gene expression by CREs have been attracting great interest [9].

In order to understand transcriptional regulation by CREs, it is necessary to investigate which parts of the nucleotide sequence of CREs are important. Effects of CRE mutations on transcriptional activity have been investigated by several approaches such as point mutation [10], saturation mutagenesis [11], and promoter shuffling [12]. Recently, a more high-throughput approach was developed [13] by combining DNA synthesis technology [14] with a programmable microarray [15] and next generation sequencers (NGS) [16]. By combining these technologies, a previous paper developed a synthetic saturation mutagenesis method to evaluate functions of mutant CREs in a high-throughput manner [13]. In the report, mutant CREs were synthesized in parallel on a programmable microarray. Each oligonucleotide was designed to have a unique sequence (a DNA barcode) downstream of the transcription start site, and used to identify individual mutant CREs. The mutated CRE library was transcribed *in vitro* and the constructed RNA library was reverse transcribed then sequenced by NGS. The relative abundance of each barcode revealed transcriptional activity of each mutant CRE. The synthetic saturation mutagenesis method has been used for analysis of various CREs, not only *in vitro*, but also *in vivo* [17]. However, this method has two issues to be improved upon. First, this method requires a special instrument, a programmable microarray, to synthesize randomized CREs. Second, it is difficult to examine multiple base substitutions. Although the synthetic saturation mutagenesis method enables the evaluation of single- and double-base substitutions, it is difficult to evaluate more than triple-base substitutions because of the difficulty in synthesizing multi-base-substituted DNAs using the programmable microarray.

Here, we developed an improved method for characterizing a variety of mutant CREs without using special instruments and in a more high-throughput manner. Our method enables simple analysis of various base-substitution patterns with relatively high coverage. We used biased randomization by PCR to obtain a CRE mutant library. In addition, we added a unique DNA barcode sequence to each CRE mutant. The constructed DNA library was transcribed *in vitro* to obtain a RNA library. Then the DNA and RNA libraries were analyzed by NGS. Based on DNA barcode information, the NGS analyses connect the information of the mutations in the CREs and the RNA abundance, which enables to explore the influences of single or multiple mutations in the CREs on transcriptional regulation.

To demonstrate our methodology, we selected the bacteriophage T7 promoter as a target CRE. The T7 promoter is a sequence of bacteriophage DNA recognized by T7 RNA polymerase [18]. The T7 promoter is used for expression of cloned genes [19] and for *in vitro* transcription [20,21] In this study, we analyzed 7847 T7 promoter variants, and quantified their transcriptional activities in a single NGS run, demonstrating the usefulness of our simple method for investigating sophisticated transcriptional regulation by CREs.

## Results

### Strategy for high-throughput evaluation of randomized T7 promoter sequences

**Fig 1:**
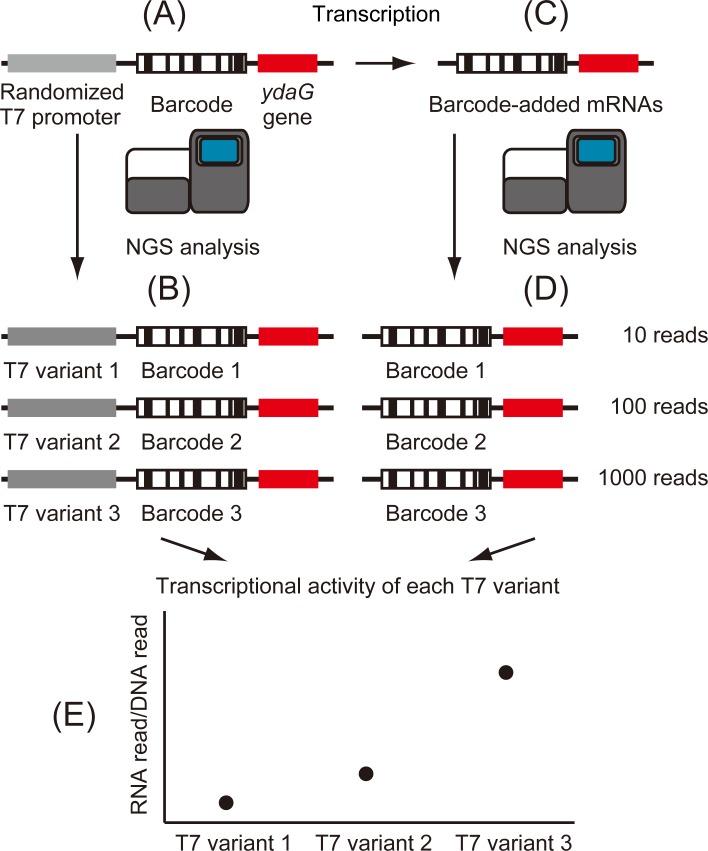
Strategy for high-throughput evaluation of randomized T7 promoter sequences. (A) Construction of DNA fragments. DNA fragments possess a randomized T7 promoter and a random 16 bases (barcode sequence) upstream of the *ydaG* gene fragment. In addition, a universal sequence was added for NGS analysis (shown in Materials and methods). (B) NGS analysis of the DNA library. Through this analysis, the relationships between barcode sequences and T7 promoter variant sequences were clarified. In addition, the read number of each DNA sequence was counted. (C) Construction of a RNA library. RNA fragments were obtained by *in vitro* transcription of the DNA library. Transcribed RNA included barcode sequences. (D) NGS analysis of the RNA library. Through this analysis, the read count of each barcode was determined by NGS. (E) Integration of NGS data. By dividing RNA-derived barcode counts by the corresponding DNA counts, the transcriptional activity of each T7 promoter variant was calculated.

In this research, we selected 19 bases of T7 promoter sequence (5’-TAATACGACTCACTATAGG-3’) as a target CRE. Transcription starts at the underlined G in the 19 bases. We designed a strategy to develop a high-throughput, universal and simple method to evaluate transcriptional activity of mutant T7 promoter variants (Fig 1). First, we constructed *ydaG* gene fragments with a biased randomized T7 promoter and a barcode sequence (Fig 1A). Barcode sequences are consecutive, defined 16 nucleotides. From constructed DNA library, 1 ng DNA fragments were used to clarify the relationships between the randomized T7 promoter sequences and the barcode sequences by NGS. In addition, DNA-seq enabled counting copy numbers of each T7 promoter variant (Fig 1B). From constructed DNA library, 800 ng DNA fragments were transcribed into mRNA by *in vitro* transcription (Fig 1C). Each transcribed mRNA contains a unique barcode sequence and *ydaG* gene sequence. These mRNAs were reversely transcribed, and the copy number of each barcode was quantified by NGS (Fig 1D). Finally, by integrating the results of DNA-seq and RNA-seq, transcriptional activity of each T7 promoter variant was evaluated.

### Preparation of DNA samples

**Fig 2:**
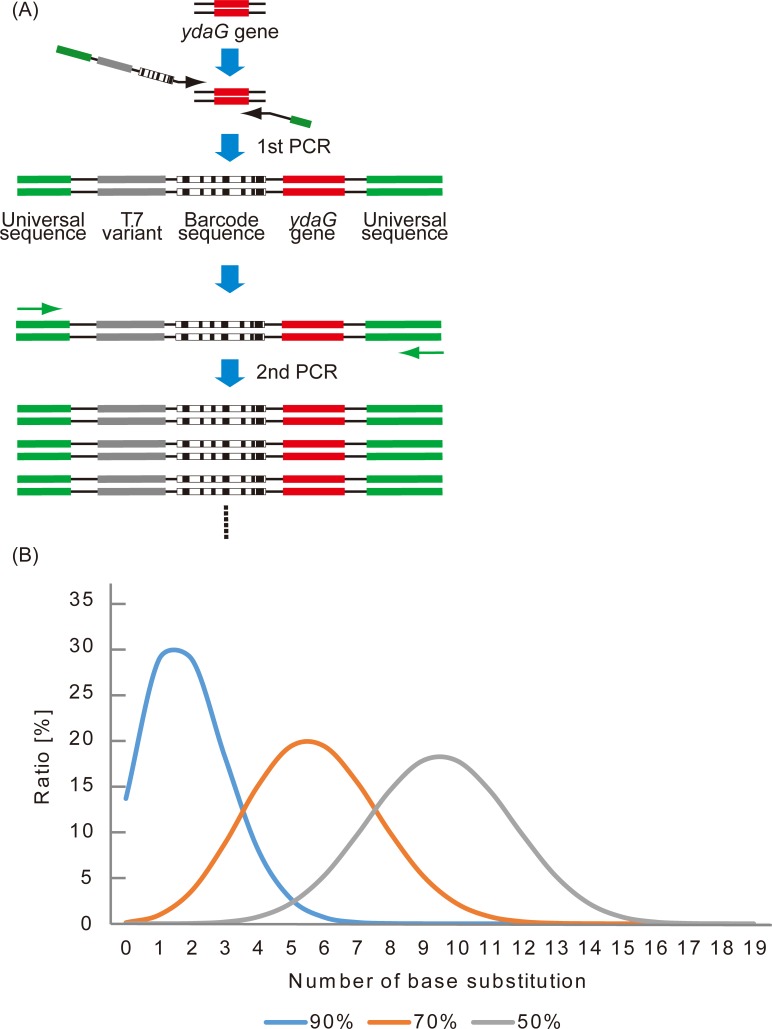
Strategy for construction of DNA and RNA samples. (A) Strategy for construction of DNA samples. DNA samples were constructed by two PCRs. The first PCR was performed to add universal sequence, a randomized T7 promoter, and a barcode sequence to the template. A second PCR was performed to amplify the DNA samples over 30 cycles. (B) Theoretical distribution of the number of base substitution for each randomization ratio. Blue line shows the retention rate at 90%. Orange line shows the retention rate at 70%. Black line shows the retention rate at 50%.

DNA fragments with randomized T7 promoter and barcode sequences were generated by two different PCRs (Fig 2A). In the first PCR, a *ydaG* gene fragment (301 bp) obtained from ASKA library [22] was used as a template. The *ydaG* gene was selected because the sequence is short and easy to manipulate. In this PCR, an adapter sequence necessary for NGS analysis, a biased randomized T7 promoter sequence, and a barcode sequence were added to the *ydaG* gene fragment. Each T7 promoter nucleotide was designed to preserve the original base at 70% probability as described in the Materials and methods. For example, if the original base was A, it was designed so that 70% remained as A, 10% as T, 10% as G, and 10% as C. The retention rate was set to 70% because we aimed to obtain a randomized T7 library in which the number of base substitutions ranged from 0 to 5. Fig 2B shows the theoretical distribution of the number of base substitutions at various retention rates as described in the Materials and methods. If the retention rate is too high (such as 90%), original T7 sequence will account for the majority (13.5%). On the other hand, if the retention rate is too low (such as 50%), T7 promoter variants with almost no transcriptional activity due to many mutations account for the majority. When the retention rate is set to 70%, the number of base substitution is distributed around 0 to 5, and the population of the original T7 promoter sequence is under 0.1%. As a DNA barcode, a randomized 16 base sequence was used. Each base was synthesized with equal probability (A = 25%, T = 25%, G = 25%, C = 25%). We used DNA barcodes with 16 bases to make the library size of the DNA barcode much larger than that of T7 promoter variants.

The primers used in the second PCR had sequences homologous to both ends of the fragments obtained in the first PCR. Therefore, DNA fragments were amplified which maintained randomized DNA sequences. The amount of DNA used as template was 0.05 pg (1.5 × 10^5^ molecules). This was because of the capacity of the MiSeq Reagent kit v3 used in this study and it can read 25 million sequences. However, exceeding the capacity of the kit causes significant decline in the read number. Therefore, we set the estimated read number as 60% of the maximum reads, i.e., 15 million in this research. In addition, we set the number of read depth coverage (times sequenced) as 100. Considering these factors, the molecular number of the template was set to 1.5 × 10^5^ molecules. The number of T7 promoter variants examined in a single NGS run was approximately the same as this number. In the second PCR, the number of amplification cycles was set to 30 to obtain a sufficient quantity of DNA for NGS analysis. Results from an Agilent 2100 Bioanalyzer platform showed that the first and second PCRs worked as expected (S1 Fig). Moreover, RNA fragments (334 nt) transcribed from DNA templates were also confirmed (S1 Fig). Based on these results, we concluded that high-quality DNA and RNA samples for NGS analysis were obtained.

### Assessment of transcriptional activity of T7 promoter variants by NGS analysis

The DNA library with randomized T7 promoter sequences and transcribed mRNA with barcode sequences was analyzed by NGS. Firstly, the quality of the DNA library was assessed. The nucleotide bias at each position of the T7 promoter sequences was about 70% as we designed (Table 1). Although the average read depth of coverage was expected to be 100, the obtained average read depth was 24.8 times. There may be several reasons for the decrease in average sequence depth. One such reason could be NGS read errors that lead to detection of false T7 sequences which did not exist in the DNA library.

**Table 1:** The nucleotide bias at each position of the T7 promoter library.

Position	Original base	G (%)	A (%)	T (%)	C (%)
-17	T	9.5	9.6	73.6	7.3
-16	A	11.5	66.6	12.9	9.0
-15	A	11.2	66.9	13.7	8.2
-14	T	9.5	9.0	73.8	7.7
-13	A	10.3	67.3	12.9	9.5
-12	C	13.2	11.7	13.7	61.3
-11	G	69.9	9.8	12.4	7.9
-10	A	11.0	66.1	13.1	9.8
-9	C	12.2	11.9	15.6	60.3
-8	T	9.6	8.9	73.9	7.6
-7	C	11.9	12.2	14.3	61.6
-6	A	10.7	66.8	13.3	9.2
-5	C	11.8	12.2	15.8	60.3
-4	T	10.1	10.4	72.3	7.2
-3	A	11.2	65.7	14.8	8.2
-2	T	10.1	11.0	72.3	6.6
-1	A	12.4	66.8	12.8	8.0
1	G	70.5	10.1	11.9	7.5
2	G	67.5	12.2	12.5	7.8

**Fig 3:**
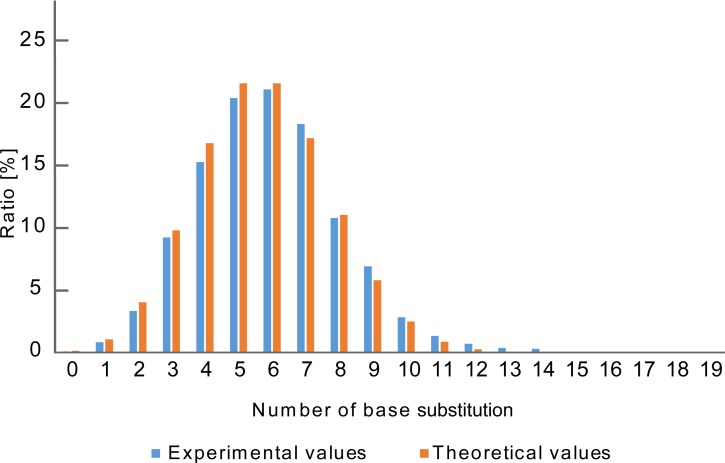
Distribution of the number of substituted bases. Distribution of the number of base substitutions. Blue means the experimental value. Red indicates theoretical values (retention rate = 70%). The method of calculation is shown in Materials and methods.

Next, the distribution of the number of base substitutions was investigated. The obtained distribution pattern roughly corresponded to the theoretical one (Fig 3). Subsequently, the coverage rate of each base substitution number was examined. As a result, almost all kinds of single and double-base substitutions were confirmed at coverage rate of 100% and 90% respectively (Table 2). Moreover, coverage rates of 32% and 20% were obtained for 3- and 4-base substitutions which were not analyzed in the previous research [13]. However, as for the 5 or more base substitutions, it was difficult to obtain sufficient coverage rate using MiSeq because of the exponentially increasing number of combinations.

**Table 2:** Coverage rate (%) of each base substitution group.

Number of base substitutions	Coverage rate [%]
1	100 (57/57)
2	90 (1385/1539)
3	32 (8493/26163)
4	20 (64289/313956)
5	3.0 (84775/2825604)
6	0.45 (88569/19779228)
7	0.068 (75282/110198556)
8	0.010 (49998/495893502)

The numbers in parentheses indicate experimental values (left) and theoretical values (right).

**Fig 4:**
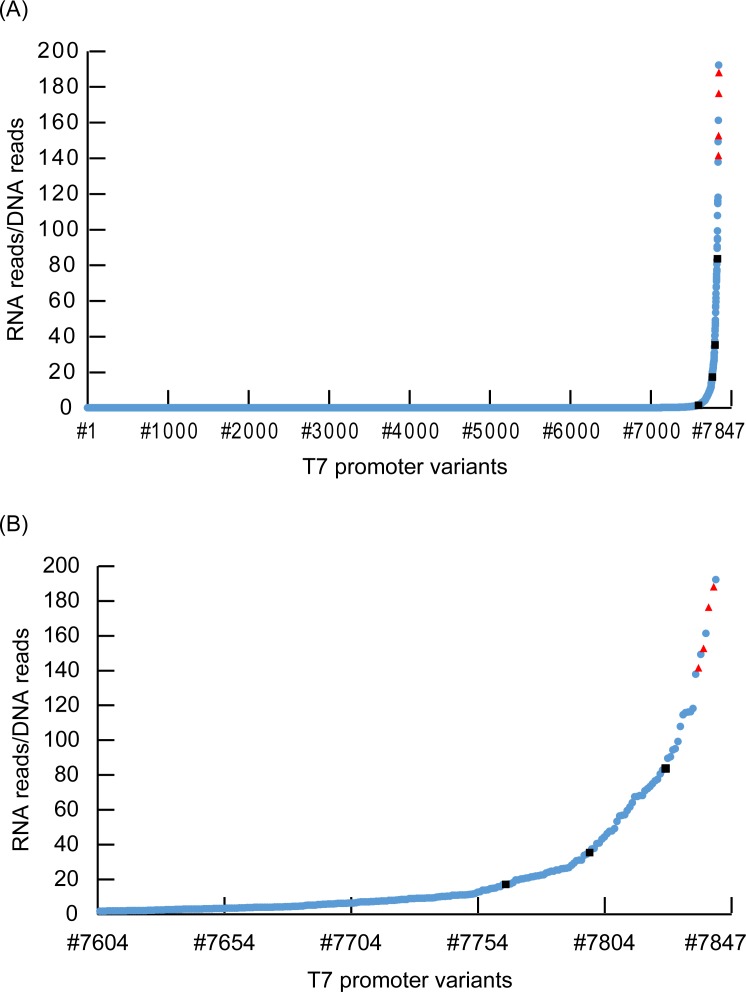
Distribution of the transcriptional activity of each T7 promoter variant sequence. (A) Distribution of all T7 promoter variants. The vertical axis means the transcriptional activity. Transcriptional activity was defined as the number of RNA reads/DNA reads. All T7 variants (from #1 to #7847) were listed in order of transcriptional activity. Blue circle plots show transcriptional activity of each randomized T7 promoter sequence. Five red triangle plots show transcriptional activity of original T7 promoter sequence with different barcodes. Four Black square plots show T7 promoter sequences used in Fig 5 experiment. There is some overlap between plots. (B) Distribution of T7 variants from #7604 to #7847.

We sought to quantify the transcriptional activity of each T7 promoter variant by integrating DNA-seq and RNA-seq data. To obtain accurate data, we focused on the T7 promoter variants with read numbers greater than 100 in DNA-seq data. Python scripts used for data analysis are presented in S1 File and the workflow is shown in S2 Fig. First, based on the data from DNA-seq, the relationship between T7 promoter variants and barcode sequences was clarified. Using the Excel COUNTIF function, we examined the duplication number of each barcode sequence. According to this analysis, it was revealed that all barcode sequences were unique ones. This result demonstrated that the sequences of the T7 promoter variants corresponded one-to-one to the barcode sequences (S2 File). Then, based on data from RNA-seq, the read number of each barcode sequence was determined. The transcriptional activity was quantified by the counts of barcode sequences. Each barcode sequence was normalized by dividing by the counts of the corresponding T7 promoter variants. As a result, we succeeded in quantification of transcriptional activity of 7847 T7 promoter variants (S2 File). Fig 4A shows the data of all T7 variants arranged in order of transcriptional activity. We found that 97% of T7 promoter variants showed relative transcriptional activity under 1% compared with the original T7 sequences. T7 promoter variants with relative transcriptional activity over 1% occupied the remaining 3% (Fig 4B and S2 File). Among the plots, the five red triangles are original T7 promoter sequences tagged with different barcodes. These five plots with the original T7 promoter sequence were confirmed to have high transcriptional activity (mean value ± SD: 169 ± 19.0). The coefficient of variation was 11.2%.

### Assessment of translational activity of T7 promoter variants *in vitr*o

**Fig 5:**
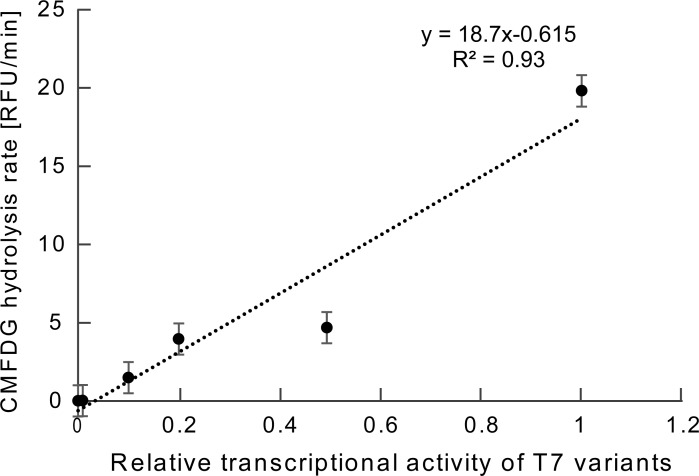
Correlation between the transcriptional activity and translational activity of *LacZ* gene with T7 promoter variant sequences. The plot illustrates the correlation between the transcriptional activity versus translational activity of LacZ production for four T7 promoter variants. Their relative transcriptional activities are 0.5, 0.2, 0.1 and 0.01. We used original T7 promoter sequence as a standard. In addition, we carried out an experiment with no DNA fragment as a negative control, and the transcriptional activity was set as zero. The translational activity was measured as the hydrolysis rate of 5-chloromethylfluorescein di-β-d-galactopyranoside (CMFDG). Each plot was averaged across three independent experiments. The error bars indicate standard errors of the means. RFU means relative fluorescence units.

We investigated whether the T7 promoters with various transcriptional activities obtained by this method were able to control the production of protein. Four different T7 promoter variants with relative transcriptional activity from 1% to 100% were randomly selected. The selected sequences were shown in S2 File. We set the number of T7 variants as four, considering the previous research [13] and the cost required for the experiment. *LacZ* gene sequence was added downstream of each T7 promoter variant by PCR. LacZ protein was synthesized using these DNA fragments within the PURE system (*in vitro* transcription and translation system) [20]. Subsequently, a fluorogenic substrate, 5-chloromethylfluorescein di-β-d-galactopyranoside (CMFDG), was added to the reaction solution. The activity of LacZ was monitored using a fluorescence microplate reader for 2 h. The initial rate of increase in fluorescence intensity is directly proportional to the amount of LacZ. Therefore, by calculating the initial rate of increase in fluorescence intensity, the relationship between the transcriptional activity of each T7 promoter variant and the amount of LacZ production was clarified (Fig 5). In this study, we did not investigate the transcriptional errors, however, the range of transcriptional errors in PURE system were not large according to a previous research [23]. Therefore, these data demonstrated that protein production can be reflected by using the T7 promoter variants obtained in this study.

## Discussion

Elucidation of the mechanisms of control of gene expression by CREs is very important not only for fundamental research in biology but also for various applications. One of the useful methods for examining CREs is synthetic saturation mutagenesis [13]. Compared with synthetic saturation mutagenesis, our proposed methodology is superior in terms of easy operation. In synthetic saturation mutagenesis, a microarray is necessary to prepare DNA fragments [14]. On the other hand, our methodology requires only a PCR thermocycler and a NGS platform. In addition, a wide range of substitution patterns can be constructed in a single sample preparation. In this study, the coverage rates of single and double nucleotide substitutions were 100% and 90%.

**Fig 6:**
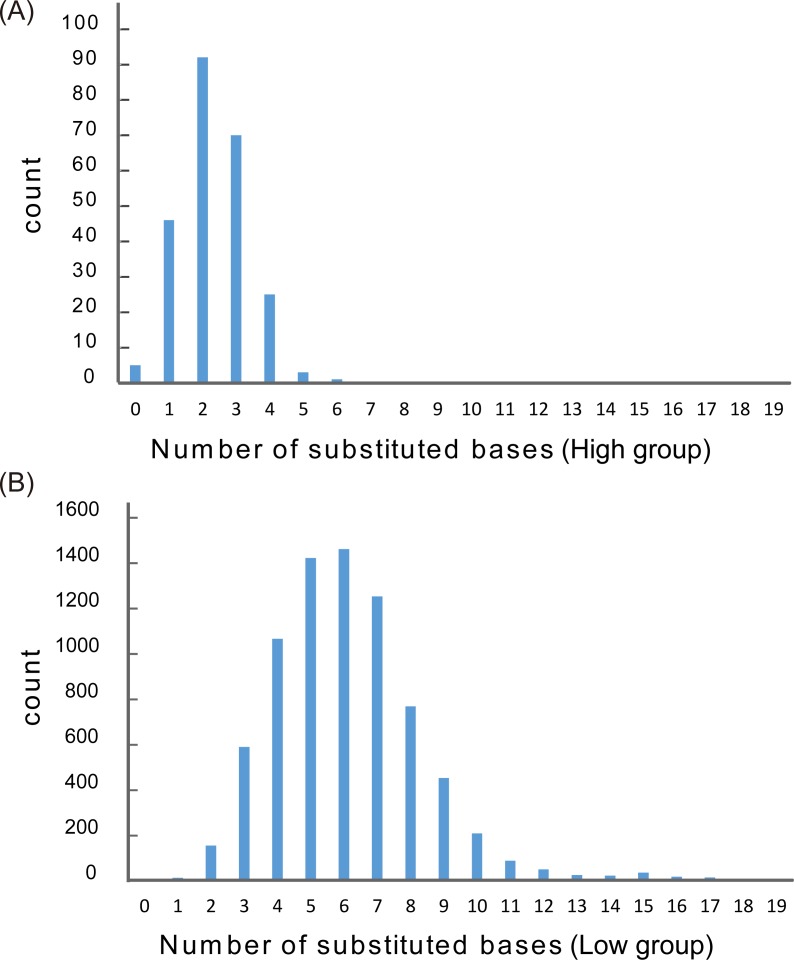
Distribution of the number of base substitution. (A) Distribution of number of substituted bases in the high group (transcriptional activity over 1% compared with original sequence). (B) Distribution of number of substituted bases in the low group (transcriptional activity under 1% compared with original sequence). Horizontal axis denotes the number of base substitutions. Vertical axis denotes the read counts.

Among the approximately 8,000 variants obtained in this study, the relative transcriptional activity of 97% of T7 promoter variants was less than 1% (low group) and the relative transcriptional activity of the remaining 3% of T7 promoter variants was over 1% (high group) (Fig 6). This figure presents a histogram of the number of base substitutions for the low and high groups. The average number of base substitutions was 2.44 for the high group and 5.97 for the low group. Also, the number of base substitutions for 87.3% of the high group was three bases or less. These results suggested that as the number of base substitution increased, the transcriptional activity decreased.

Next, we examined to what extent the original bases were conserved in the high group. For 244 T7 promoter variants in the high group, the retention rate of each base in the 19 bp sequence TAATACGACTCACTATAGG (−17 to +2) was examined. Based on the results, the nucleotides CTC (from −9 to −7) were well-preserved among the 19 bases, with a retention rate over 98% (S1 Table). The importance of this CTC sequence has been reported in previous studies, and mutations in this region are known to lower the transcriptional activity to less than 5% of the original sequence [24]. The reason why substitutions at these positions are not tolerated is that this region is involved in critical interactions between the T7 RNA polymerase and promoter. According to previous research, N748 of T7 RNA polymerase makes contacts with the base pairs at −10 and −11 [25], R756 interacts with the base pair at −9 [26], Q758 interacts with the base pair at −8 [26]. Previous studies involving X-ray crystallography have clarified that the specificity loop of RNA polymerase (residues 739 to 770) recognized the T7 promoter by interacting with the DNA major groove from –11 to −7 [27]. Single base substitutions have a great impact on the specificity of RNA polymerase for promoter sequences in general [28].

On the other hand, some members of the low group had conserved CTC sequences, yet showed very little transcriptional activity. Among these, 338 T7 promoter variants with between 0 and 3 base substitutions were obtained. The most frequent mutation involved the C at base position −5. Approximately 28% of 338 T7 promoter variants had mutations at this nucleotide. The base pair at −5 is the boundary between the binding region and the initiation region [27], and DNA melting starts from this position [28]. It was clarified in a previous study [24] that mutations at this site also significantly diminish transcriptional activity.

As stated above, these results obtained in this study coincide with the previous studies [24,28]. Therefore, we concluded that the usefulness of our novel method was verified strongly.

In order to confirm the intensity of T7 variants obtained in this study, the effect of T7 promoter variants on translation activity was evaluated by the production of protein employing an *in vitro* transcription/translation system (PURE). As a result, the transcriptional activity was found to correlate to the translational activity. From this result, it is suggested that in order to increase the production level of a protein by X fold, T7 promoter variants having a transcriptional activity X fold should be selected. The transcriptional errors of native T7 sequences (coefficient of variation = 11.2%) shown in Fig 4 was larger compared with the range of translation errors of native T7 sequences (coefficient of variation = 6.13%) shown in Fig 5. Difference in barcode sequences could increase the transcriptional errors in the experiment of Fig 4. These barcode sequences were removed in the experiment described in Fig 5, and this was likely to decrease the range of translational error bars. One reason of the outlier (the plot of transcriptional activity = 0.5) is that the transcriptional activity contains errors due to the influence of barcode sequences. Previous researches confirmed that DNA concentration correlates linearly with protein production levels under *in vitro* conditions [29,30]. Moreover, transcriptional activity of the T7 variant *in vitro* and the results of quantification by luciferase assay *in vivo* were in direct proportion [13]. Therefore, the results obtained in this study appear reasonable, given the previous studies[13,29,30].

In recent years, research involving artificial cells has received attention [31,32]. Methods to engineer gene expression similar to that in living systems under *in vitro* conditions have been developed [33,34]. In this study, we devised and demonstrated a simple and high-throughput analytical method for evaluation of mutated CRE transcriptional activity *in vitro*. Therefore, as a novel method for the investigation of gene expression involving CREs, this methodology has the potential to play an important role in the field.

## Materials and methods

### Preparation of DNA samples

Randomized T7 promoter sequences with barcode ‘tags’ were constructed *via* two PCRs. The first PCR was performed using a linear *ydaG* gene fragment (301 bp) obtained from ASKA library [22] as a template. The primers, 1-forward (5’- TCGTCGGCAGCGTCAGATGTGTATAAGAGACAGGGCCTAATACGACTCACTATAGGATAGATTCAATTGTGNNNNNNNNNNNNNNNNAGCGGATAACAATTTCACACAGAATTCAT-3’ underline means biased randomized T7 promoter; N means randomized base.) and 1-reverse (5’-GTCTCGTGGGCTCGGAGAT-3’) were used to add randomized T7 promoter and barcode sequences to the 5’ end of the *ydaG* gene. Primers were synthesized commercially by solid phase synthesis (FASMAC, Kanagawa Japan). The underlined 19-base sequence indicates the T7 promoter. These underlined bases were synthesized by biased randomization so as to retain the original sequence with a probability of 70% (e.g., A means A: 70%, T: 10%, G: 10%, C: 10%). The reason we set this retention rate value was to control the number of base substitutions around 0 to 5. We defined the distribution of T7 variants by the number of base substitutions based on the following formula. In this formula, x represents the retention rate [%] and y indicates the number of base substitutions.

$$\frac{19!}{\left(y!(19-y)!\right)}\times {\left(\frac{x}{100}\right)}^{19‑y}\times {\left(\frac{100‑x}{100}\right)}^{y}$$

According to calculations based on this formula, we set x = 0.7 as the retention rate for obtaining target T7 promoter variant patterns.

The character N in the primer sequence means a randomized base (A: 25%, T: 25%, G: 25%, C: 25%). The sequences of 16 consecutive Ns function as barcode ‘tags’. In one assay, 1.51 × 10^5^ kinds of T7 promoter variants are assessed. On the other hand, theoretically, 4.29 × 10^9^ kinds of barcode sequences are created. It is considered that the possibility of barcode duplication is very low.

The ends of both primers have sequences for the adapter necessary for the NGS analysis. Components of the first PCR mixture: 10 μL distilled water, 12.5 μL KAPA HiFi Hot Start Ready Mix (KAPA Biosystems, Wilmington, MA, USA), 0.75 μL of 1 pM each primer, 1 μL of 10 ng/μL template *ydaG* DNA. Cycling parameters: 95°C (2 min)/98°C (20 sec)/75°C (15 sec), and final hold at 4°C.

A second PCR was performed using dilutions of the first-round PCR products as template. The primers, 2-forward (5’-TCGTCGGCAGCGTCAGATGTGTATAAGAGACAGGGCC-3’) and 2-reverse (5’-GTCTCGTGGGCTCGGAGATGTGTATAAGAGACAGGTTATTGCTCAGCG-3’) were used to amplify the DNA fragments. Primers were synthesized commercially by solid phase synthesis (FASMAC). Components of the second PCR mixture were: 10 μL distilled water, 12.5 μL KAPA HiFi Hot Start Ready Mix, 0.75 μL of 10 pM each primer, 1 μL of 0.05 pg/μL template linear DNA solution. Cycling parameters: 95°C (2 min), then 30 cycles 98°C (20 sec)/75°C (15 sec)/72°C (3 min), and final hold at 4°C. The amplified DNA fragments were purified using AMPure XP magnetic beads (Beckman Coulter, Brea, CA, USA) according to manufacturer’s protocol and analyzed on an Agilent Bioanalyzer 2100 platform (Agilent Technologies, Richardson, TX, USA). The resulting DNA fragments with randomized T7 and barcode sequences were used for *in vitro* T7 transcription.

### Preparation of RNA samples

Transcribed RNA sequences were obtained using T7 transcription kit (JENA BIOSCIENCE, Jena, Germany). The *in vitro* transcription reaction solution was prepared by mixing 48 μL T7 transcription kit solutions (34.5 μL nuclease-free water, 10 μL 5 × T7 reaction buffer, 2.5 μL 10 mM dNTP mix, 0.5 μL 40 units/μL RNase inhibitor, and 0.5 μL 200 units/μL T7 RNA polymerase) and 2 μL 400 ng/μL template DNA. The reaction solution was incubated at 37°C for 60 min. After incubation, 1 μL 5 U/μL recombinant DNase I (Takara Bio, Kusatsu, Japan) was added and the reaction solution was incubated at 37°C for 15 min. RNA fragments were extracted using RNeasy Mini Kit (QIAGEN, Hilden, Germany) according to manufacturer’s protocol. RNA fragments were analyzed and quantified by Agilent Bioanalyzer 2100.

### Illumina sequencing and data analysis

Preparation of DNA sample for NGS was carried out by PCR to add index sequences to the DNA fragments. Components of PCR mixtures included: 10 μL distilled water, 25 μL KAPA HiFi Hot Start Ready Mix, 5 μL Nextera XT Index primer 1 (Illumina, San Diego, CA, USA), 5 μL Nextera XT Index primer 2 (Illumina), and 5 μL 10 ng/μL DNA fragment as template. Cycling parameters: 95°C (3 min), then 8 cycles 98°C (30 sec)/55°C (30 sec)/72°C (30 sec), 72°C (5 min), and final hold at 4°C. The amplified DNA fragments were purified using AMPure XP magnetic beads, according to manufacturer’s protocol. Next generation sequencing of DNA samples was done using MiSeq Reagent Kit v3 150 cycles (Illumina). Preparation of RNA sample for NGS was carried out using KAPA Stranded mRNA-Seq Kit (KAPA Biosystems) according to the kit protocol. RNA samples were analyzed by NGS. MiSeq Reagent Kit v3 150 cycles (Illumina) was used.

Data were analyzed using a Python script shown in S1 File. Using DNA fastq file and Python scripts (DNAcount.py and DNAextract.py), T7 promoter variants and barcode sequences were extracted and the numbers of reads were counted. Target average sequencing depth varies depending on types of researches [35]. In this research, there is a concern for decreasing data quality due to the sequencing errors. Therefore, we initially designed an experimental plan to obtain 100 reads of each DNA sequence, but the average depth obtained from our experiment was 24.8. Hence, we set the cut off for analysis at 100 according to the initial plan to avoid decreasing data quality. Using RNA fastq file and Python scripts (RNAcount.py and RNAextract.py), barcode sequences were extracted and the number of each barcode sequence was counted. Finally, using Python script bar_T7_read_ratio.py, DNA data and RNA data were integrated. Based on these data, transcriptional activity (read number of RNA/read number of DNA) was calculated. The processed data are presented in S2 File. The raw NGS data are available from the NCBI database (NCBI SRA accession: SRP127515).

### *In vitro* transcription and translation for evaluation of T7 promoter variants

To assess the effect of T7 promoter variants on translation, DNA fragments with T7 promoter variants were constructed. Assessed T7 promoter variants were selected randomly from sequences with relative transcriptional activity from approximately 1% to 100% compared to the original T7 promoter. *LacZ* gene sequences were added downstream of selected T7 promoter variants by PCR. Primers used to amplify the DNA fragments were shown in S2 Table. Primers were synthesized commercially by solid phase synthesis (FASMAC). The plasmid coding *lacZ* gene sequence (obtained from ASKA library [22]) was used as a template. Components of PCR mixture included: 32 μL distilled water, 5 μL PCR buffer for KOD-Plus-Neo buffer, 3 μL 25 mM MgSO_4_, 3 μL 2 mM dNTPs, 1.5 μL 10 pmol/μL forward primer, 1.5 μL 10 pmol/μL reverse primer, 1 μL of template ASKA 10 ng/μL, 1 μL of KOD-Plus-Neo DNA polymerase (TOYOBO, Tokyo, Japan). Cycling parameters: 94°C (2 min) then 30 cycles 98°C (10 sec)/45°C (30 sec)/68°C (2 min), 68°C (3 min), and final hold at 4°C. An *in vitro* transcription and translation reaction solution, PUREfrex*®*2.0 (GeneFrontier, Kashiwa, Japan) was used. The PURE system [20] contained all factors for transcription and translation. T7 RNA polymerase was adopted for transcription in this PURE system. 10 ng/μL DNA fragments were mixed with the PURE system solution and incubated at 37°C for 60 min. After incubation, 1 μL of 200 μM kanamycin was added to terminate production of LacZ protein. In addition, 1 μL 500 μM CMFDG (Life Technologies, Carlsbad, CA, USA) was added as the fluorogenic substrate. CMFDG has two galactose moieties and is a sensitive substrate for LacZ activity. Hydrolysis by LacZ can be monitored by the fluorescence produced from CMFDG. The reaction solution was incubated at 37°C for 2 h. Fluorescence was monitored every 30 sec at λ_ex_ = 485 nm and λ_em_ = 510 nm using a Fluoroskan Ascent FL system (Labsystems, Helsinki, Finland).

## Supporting information

S1 File Python scripts for data analysis. (DOCX)

S2 File Read number of each T7 promoter and barcode sequence. This table shows the read number of each T7 promoter variant and barcode sequence. This table also presents the transcriptional activity (RNA read/DNA read) of each T7 promoter variant.
(XLSX)

S1 Fig Results of micro-capillary electrophoresis. (A) Results of micro-capillary electrophoresis of DNA samples using Agilent Bioanalyzer 2100. The peaks at 35 bp and 10,000 bp indicate size markers (M). The peak around 400 bp shows the target sequence. FU represents fluorescence units. Horizontal axis shows the electrophoretic mobility. (B) Results of micro-capillary electrophoresis of RNA samples. The peak at 25 nt is a marker (M). The peak around 400 nt shows the target RNA. FU indicates fluorescence units. Horizontal axis shows the electrophoretic mobility.
(EPS)

S2 Fig Work flow of NGS data analysis. This figure shows the procedure of how to analyze the data obtained from NGS.
(EPS)

S1 Table Retention rate of each base of T7 promoter variants. This table shows the retention rate of each base of 244 T7 promoter variants in the high group.
(XLSX)

S2 Table Primers to construct DNA fragments with T7 variants and *lacZ* gene. This table shows the primer list for construction of DNA fragments used for translational assay in PURE system.
(XLSX)
