# Supplementary material for: High-throughput evaluation of T7 promoter variants using biased randomization and DNA barcoding
Source: PLoS One. 2018 May 7;13(5):e0196905. doi: 10.1371/journal.pone.0196905 (PMC5937735; doi:10.1371/journal.pone.0196905)
Supplement: S1 File — (DOCX) [file pone.0196905.s001.docx]

- DNAextract.py

f = open("DNA.fastq")

f2 = f.readlines()

count = 0

for l in f2:

count += 1

if count % 4 == 2:

if "AGCGGATAACA" in str(l):

l = str(l).split("AGCGGATAACA")

wanted = l[0][-50:]

if len(wanted) == 50:

splited = wanted.split("ATAGATTCAATTGTG")

if len(splited) == 2:

if len(splited[1]) == 16:

print wanted

#The purpose of this script is the extraction of the correct sequence.

#DNA.fastq means the integrated output files.

#The processed data was saved as DNA_bar16.txt.

- DNAcount.py

from collections import Counter

f = open("DNA_bar16.txt")

f2 = f.readlines()

base_list = []

for l in f2:

l = l.strip()

base_list.append(l)

counter = Counter(base_list)

for word, cnt in counter.most_common():

if cnt >= 100:

print word, cnt

#The purpose of this script is to count the copy number of each sequence.

#The sequence data counted over 100 times were saved as DNAcount_bar16_over100.fasta.

- RNAextract.py

f = open("RNA.fastq")

f2 = f.readlines()

count = 0

for l in f2:

count += 1

if count % 4 == 2:

if "AGCGGATAACA" in str(l):

l = str(l).split("AGCGGATAACA")

wanted = l[0][-16:]

if len(wanted) == 16:

print wanted

#The purpose of this script is the extraction of the correct sequence.

#RNA.fastq means the integrated output files from RNA-seq.

#The processed data was saved as RNA16.txt.

- RNAcount.py

from collections import Counter

f = open("RNA16.txt")

f2 = f.readlines()

base_list = []

for l in f2:

l = l.strip()

base_list.append(l)

counter = Counter(base_list)

for word, cnt in counter.most_common():

print word, cnt

#The purpose of this script is to count the copy number of each barcode sequence.

#The counted data were saved as DNAcount_bar16_over100.fasta.

- bar_T7_read_ratio.py

f = open("DNAcount_bar16_over100.fasta")

f2 = f.readlines()

dict = {}

dict2 = {}

for l in f2:

l = l.split()

barcode = l[0][-16:]

T7 = l[0][:19]

if barcode not in dict:

dict[str(barcode)] = int(l[1])

else:

dict[str(barcode)] += int(l[1])

if T7 not in dict2:

dict2[str(barcode)] = T7

f.close()

f3 = open("RNAcount.fasta")

f4 = f3.readlines()

for l3 in f4:

l4 = l3.split()

if l4[0] in dict:

ratio = float(l4[1])/dict[l4[0]]

print l4[0], dict2[l4[0]], dict[l4[0]], l4[1], ratio

#The purpose of this script is to link T7 promoter sequence data and RNA-seq data.

#The number of barcode sequence was devided by the corresponding DNA copy number.
